# Genome-wide identification of AGO18b-bound miRNAs and phasiRNAs in maize by cRIP-seq

**DOI:** 10.1186/s12864-019-6028-z

**Published:** 2019-08-16

**Authors:** Wei Sun, Dong Chen, Yaqiang Xue, Lihong Zhai, Dan Zhang, Zheng Cao, Lei Liu, Chao Cheng, Yi Zhang, Zuxin Zhang

**Affiliations:** 1 College of Agriculture and Biology, Zhongkai University of Agriculture and Engineering, Guangzhou, 510225 People’s Republic of China; 2 National Key Laboratory of Crop Genetic Improvement, Huazhong Agricultural University, Wuhan, 430070 People’s Republic of China; 3 Center for Genome Analysis, ABLife Inc, Optics Valley International Biomedical Park, Building 9-4, East Lake High-Tech Development Zone, 388 Gaoxin 2nd Road, Wuhan, 430075 Hubei China; 4 Laboratory for Genome Regulation and Human Health, ABLife Inc, Optics Valley International Biomedical Park, Building 9-4, East Lake High-Tech Development Zone, 388 Gaoxin 2nd Road, Wuhan, 430075 Hubei China; 5 Medical College of Hubei University of Arts and Science, Xiangyang, 441053 People’s Republic of China

**Keywords:** Argonaute, microRNA, phasiRNA, cRIP-seq, Shoot apical meristem

## Abstract

**Background:**

Argonaute proteins (AGOs) are important players in the regulation of plant development by directing sRNAs to target mRNAs. In maize (*Zea mays*), AGO18b is a tassel-enriched and grass-specific AGO. Previous studies have shown that AGO18b is highly expressed in tassels during meiosis and negatively regulates determinacy of spikelet meristems. However, binding profile on RNAs and acting mechanisms of AGO18b remain unknown.

**Results:**

In this study, we explored the binding profile of AGO18b in maize tassel by UV cross-linking RNA immunoprecipitation, followed by deep sequencing of these cDNA libraries (cRIP-seq), and systematically studied AGO18b-associated small RNAs and mRNAs by bioinformatics analysis. By globally analyzing the phased small-interfering RNA (phasiRNA) and miRNA abundance bound by AGO18b, we found AGO18b primarily binds to 21-nt phasiRNAs/miRNAs with a 5′-uridine and binds less strongly to 24-nt phasiRNAs with a 5′-adenosine in the premeiotic tassels. The abundance profile of AGO18b-associated miRNAs was different from their expression profile. Moreover, AGO18b strongly binds to *miR166a-3p*. We then obtained the AGO18b-bound mRNA targets of *miR166a-3p* by cRIP-seq, and confirmed the molecular function of AGO18b in regulating spikelet meristems.

**Conclusions:**

Our results indicated that AGO18b binds to phasiRNAs with obvious 5 prime end bias under different sRNA length. MiRNAs and their target mRNAs associated with AGO18b indicated the molecular mechanisms of AGO18b as a negative regulator of inflorescence meristem and tassel development through integrating both phasiRNAs and miRNA pathways, which extended our view of sRNA regulation in flower development and provided potential methods to control pollination in the future.

**Electronic supplementary material:**

The online version of this article (10.1186/s12864-019-6028-z) contains supplementary material, which is available to authorized users.

## Background

In eukaryotes, Argonaute (AGO) proteins could regulate the stability and translation level of bound mRNAs by facilitating the base pairs between small RNAs (sRNAs), including miRNAs, and target mRNAs [1–4]. AGOs and sRNAs, as well as other proteins, forming a ribonucleoprotein called RNA-induced silencing complex (RISC), play important roles in post transcriptional regulation [4, 5]. In plants, phylogenetic analysis of AGO proteins from *Arabidopsis* [4], rice [6], soybean [7] and maize [8], grouped AGOs into three major clades: AGO1/5/10, AGO2/3/7, and AGO4/6/8/9 [9]. The biological functions of some AGO proteins have been well studied by identifying their binding RNAs using immunoprecipitation and/or high throughput sequencing methods [10, 11]. In *Arabidopsis*, two AGO proteins, AGO1 and AGO10, have been shown to regulate the development of shoot apical meristem (SAM) [11–17] and of floral stem cells [18] through binding miR165/166 and thus triggering the downstream pathway.

Maize (*Zea mays*) is one of the most important economical crops in the world. There are two types of inflorescences in maize: male inflorescence (tassel) and female inflorescence (ear) [19]. During embryogenesis, the terminal apical meristem elaborates the tassel, while one or more meristems in the axils of vegetative leaves develop as ears [20]. Based on the extensively studied molecular mechanism of reproductive transition and the determination of meristem identity [21–23], recent studies have revealed that miRNAs could regulate the reproductive transition by repressing the expression of related key TFs, forming post-transcriptional and transcriptional regulatory networks. For example, in *Arabidopsis*, *miR156* and *miR172*, and their respective targets form a regulatory axis to control the reproductive transition and flowering [23–25]. *MiR156* could regulate the expression of *miR172* via targeting *SPL9* [25], while *miR172* represses the translation of another TF gene, *APETALA2* (*AP2*), whose protein controls the flower development [24, 26, 27]. In grass species, the regulatory axis of *miR156, miR172* and their targets affects multiple aspects of development, from leaf and tiller initiation to floral organ differentiation [28–30].

In our previous study, we have systematically validated 17 AGO genes in maize by bioinformatics and rapid amplification of cDNA ends (RACEs) method [8]. We found that both maize *AGO18a* and *AGO18b*, a deep branch in AGO1/5/10 clade, are highly expressed in tassels, and *AGO18b* mRNA is enriched in the tapetum and germline cells during meiosis [8]. A recent study showed that repressing AGO18b expression leads to an increased number of spikelets on the central spike, and AGO18b may function in tassel development by interacting with the miR166-mediated regulatory pathway [31]. However, the molecular mechanism of how AGO18b regulates the tassel development is not clear.

Interestingly, recent studies also showed that 21-nt and 24-nt phased small-interfering RNAs (phasiRNAs) may play an important role in the grass inflorescence development [32, 33]. Production of these two classes of phasiRNAs both require miR2118 and miR2275, RDR6, and dicer proteins [34, 35]. *MEL1/OsAGO5c* can bind to 5′-cytosine of 21-nt phasiRNAs that are abundant in rice genome, and are preferentially expressed in developing inflorescences [36, 37]. A recent study on maize anthers has identified a set of loci encoding 21-nt and 24-nt phasiRNAs, and has found that the 21-nt phasiRNA population is predominantly expressed during the premeiotic stage while the 24-nt population is expressed in anther at the meiotic stage [38]. The spatial location of the 24-nt phasiRNAs in anthers matches that of the AGO18b, suggesting AGO18b acts as the partner of the 24-nt phasiRNAs [38].

In this study, we systematically studied AGO18b-bound sRNAs and mRNAs in the premeiotic tassels by UV cross-linking RNA immunoprecipitation (cRIP) using an antibody against the endogenous AGO18b protein, followed by deep sequencing of these cDNA libraries. We found that AGO18b preferentially binds sRNAs and phasiRNAs with distinct features, and strongly associates with maize miR166a and its target mRNAs. Finally, we proposed that AGO18b interacts with the miR166-HD-ZIP III TF regulatory pathway and regulates IM development of maize tassel.

## Results

### AGO18b preferentially associates with 21-nt phasiRNAs in pre-meiotic tassels

**Fig. 1:**
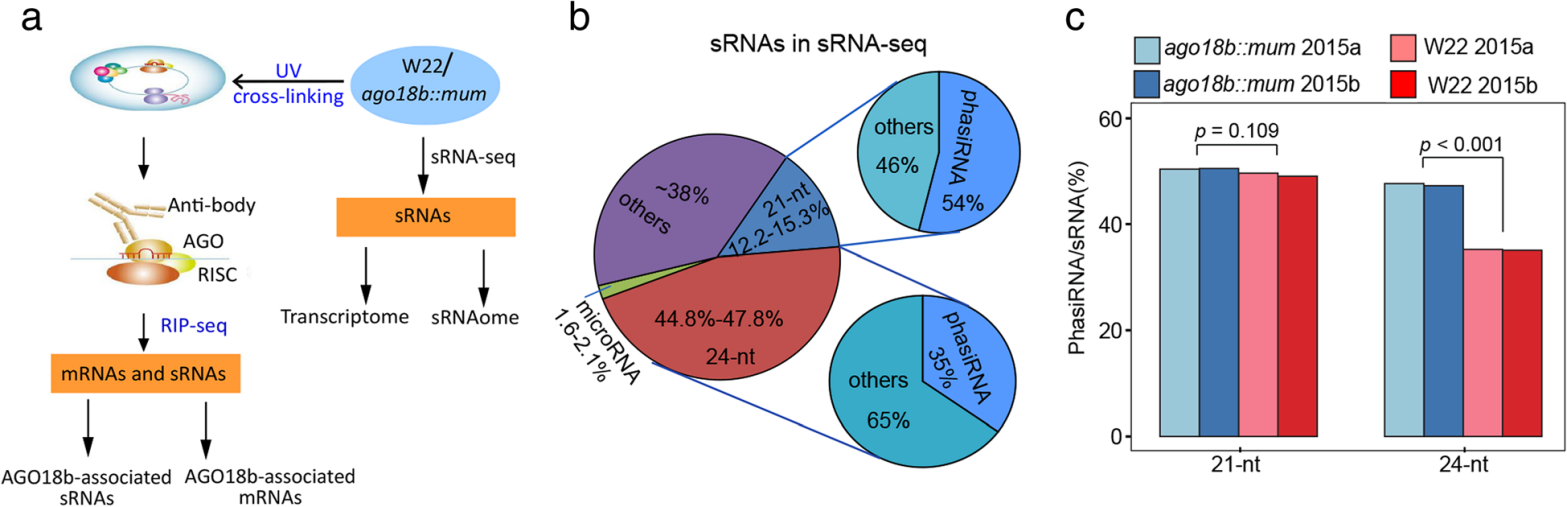
The flowchart of experiments and expression profile of captured sRNAs in WT and *ago18b::mum* samples. **a** Flowchart showing the experiments and analysis strategies used in this study. **b** Pie chart showing the average percentages of different small RNAs in sRNA-seq libraries from two independent biological replicates. **c** Bar plot showed that the percentages of 24-nt phasiRNAs in small RNAs were significant different between W22 and ago18b::mum samples (*p*-value < 0.001, t-test)

Considering that AGO18b mediates regulation of inflorescence meristem probably by interacting with miR66-HD-ZIP III TF regulatory pathway [31], we decided to re-study the expression profile of small RNAs (sRNAs) in the 6-cm stage tassels of wild-type (W22-ref) and Mutator-mediated mutant of *ago18b* (*ago18b::mum*, Uniform Mu01249) plants from the small RNAs sequencing data (sRNA-seq) [31] (Fig. 1a). These two replicate sequencing samples were obtained with same experimental procedures and can be treated as biological replicates. Re-analyzing the sRNA-seq data from the 6-cm tassels revealed that 24-nt sRNA was the predominant population (45–48%), 21-nt population was the second (12–15%), and microRNAs constituted just a little percentage of all the sRNAs (Fig. 1b, Additional file 1: Figure S1a, and Additional file 2: Table S1 and S2). Subsequently, we analyzed the phasiRNAs in these sRNA-seq datasets by mapping onto the recently identified 21-nt and 24-nt phasiRNA loci [38] (Additional file 2: Table S3). Overall, there were more expressed 24-nt than 21-nt phasiRNAs in maize tassels (Additional file 1: Figure S1b). In the W22-ref tassel, ~ 54% of the 21-nt sRNAs were mapped onto phasiRNA loci, while ~ 35% of the 24-nt sRNAs were mapped onto phasiRNA loci (Fig. 1b). Therefore, the 21-nt phasiRNA fraction in the 6-cm tassel (~ 0.3-mm anther) closely resembles that of the anther in its 0.4-mm premeiotic stage [38]. However, the fraction of the 24-nt phasiRNA identified in this study differed greatly from that in anther (phasiRNAs/sRNA = 1–2% in anther) [38], indicating that 24-nt phasiRNAs were enriched in floral organs other than anther. In the *ago18b::mum* tassel, a significant proportional decrease of 24-nt phasiRNAs was observed compared to that in W22-ref, while the 21-nt phasiRNAs showed no difference (Fig. 1c). These results indicated that 24-nt phasiRNAs were enriched in floral organs other than anther and their production may be regulated by AGO18b.

**Fig. 2:**
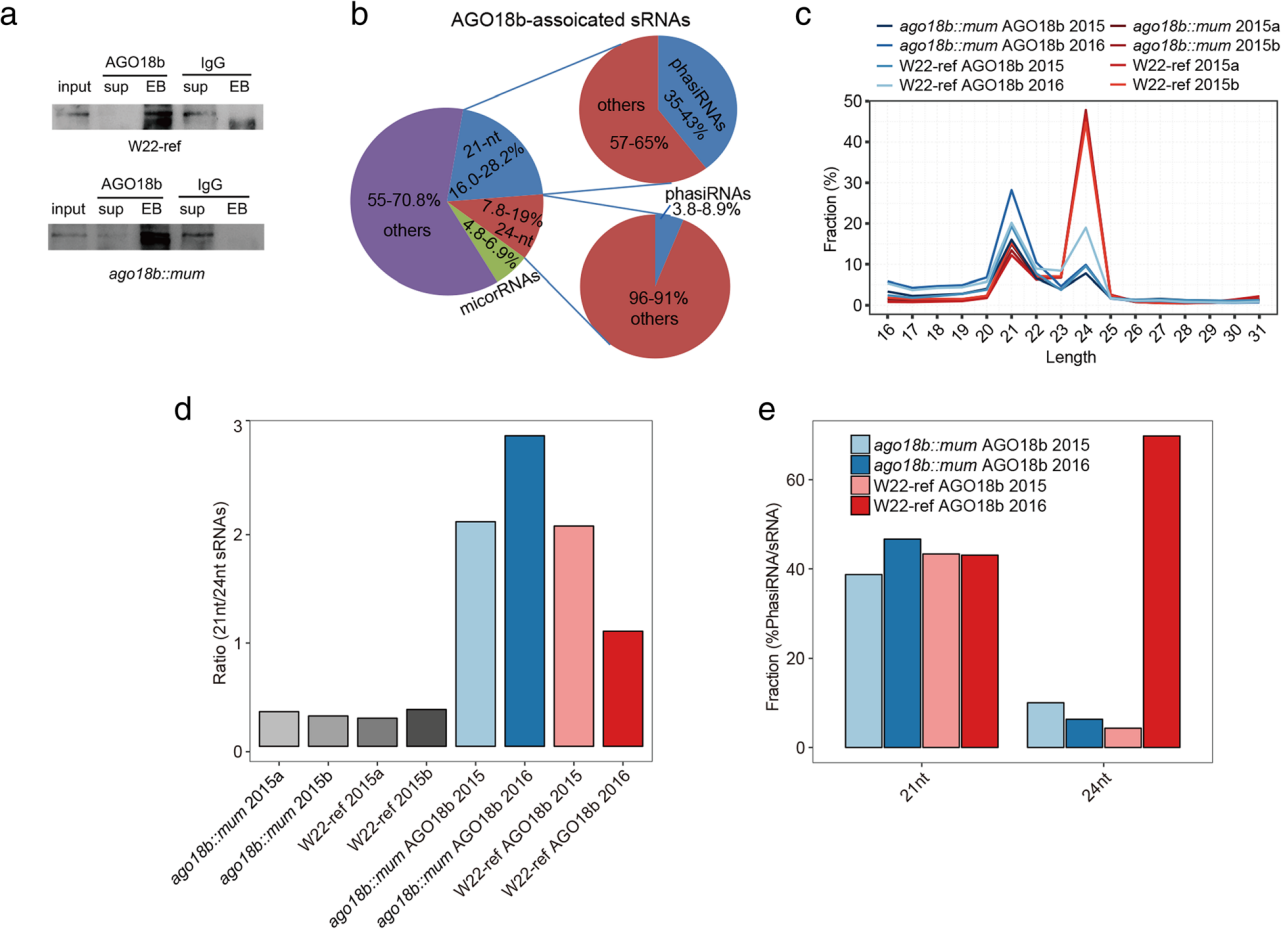
The features of AGO18b-bound sRNAs and total sRNAs by cRIP-seq and sRNA-seq, respectively. **a** Western blot experiment of AGO18b and IgG for W22-ref samples (top) and *ago18b::mum* samples (bottom). **b** Pie chart showing the average percentages of different sRNAs in RIP-seq libraries from two independent biological replicates. **c** Line plot showing the length distribution of sequenced sRNA reads in sRNA-seq libraries and AGO18b-bound sRNA libraries. The 21-nt sRNAs were preferred bound by AGO18b in cRIP-seq samples. **d** Bar plot showing the ratio between 21-nt and 24-nt sRNAs in the sRNA libraries and AGO18b-bound sRNA libraries. **e** Bar plot showing the percentages of 21-nt and 24-nt phasiRNAs in AGO18b-bound sRNA libraries

As previously described, the Mutator-mediated mutant resulted in a reduction of AGO18b protein level in the developing tassels of *ago18b::mum* relative to that in W22-ref [31]. We used a modified RNA immunoprecipitation (RIP) method to identify AGO18b-bound sRNAs in both W22-ref and *ago18b::mum* samples (Fig. 1a). AGO18b protein was cross-linked by UV irradiation with its bound RNAs. The cross-linked protein and RNA complex were then immunoprecipitated by antibody specifically against AGO18b using IgG as control (Fig. 2a). The cDNA populations showing a distinct difference between AGO18b antibody-precipitated samples and IgG-precipitated samples in their intensities on a gel (Additional file 1: Figure S2a-b)**.** In order to eliminate the variation between plants and experiments, analysis was performed separately on 6-cm tassels grown in 2015 and 2016. The AGO18b-bound samples showed much higher percentage than the IgG samples for filtered reads, especially for the experiment using 2015-grown tassels (Additional file 2: Table S1). Notably, AGO18b-bound sRNAs showed a drastic decrease in the 24-nt population in both W22-ref and *ago18b::mum* tassels, accompanied by an increase of 21-nt sRNAs, as compared to their proportions in the sRNA-seq (Figs. 1b, 2b-c). In three of the four wild-type and mutant samples, the 21-nt population increased to a higher proportion than the 24-nt (Fig. 2d). These results suggested that AGO18b preferentially binds to 21-nt sRNAs in maize premeiotic tassels. Importantly, about 35–43% of AGO18b-bound 21-nt sRNAs could be mapped to phasiRNA loci in the four sequenced samples (Fig. 2e). This result was supported by the heatmap analysis (Additional file 1: Figure S2c-d). However, only 3.8–8.9% of AGO18b-associated 24-nt sRNAs could be mapped to 24-nt phasiRNAs in three experiments, with one exception (Fig. 2e). These results suggest a strong affinity of AGO18b for 21-nt phasiRNAs.

### AGO18b preferentially binds to 21-nt sRNAs/phasiRNAs with a 5′-uridine and 24-nt sRNAs/phasiRNAs with a 5′-adenine

**Fig. 3:**
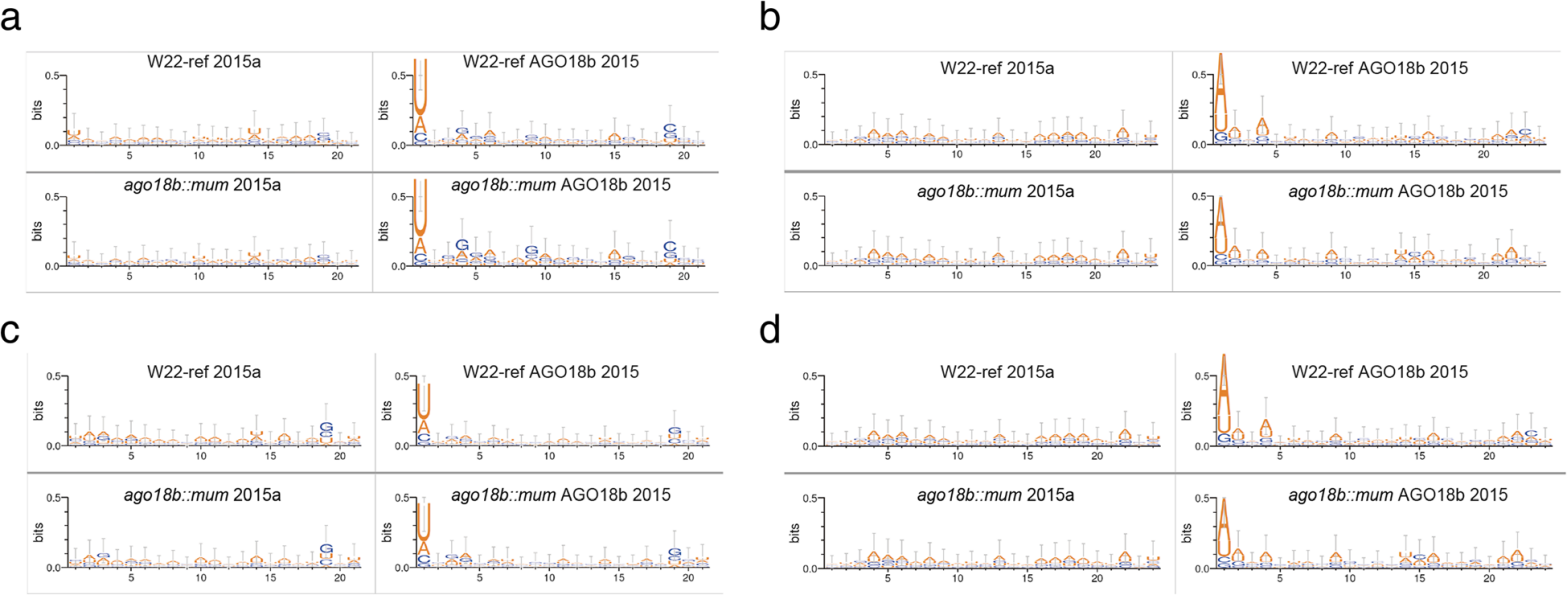
The 5′ base composition analysis of sequenced sRNAs from cRIP-seq and sRNA-seq. **a-d** Base composition of reads from RNAome sRNAs libraries (Left, each panel) and AGO18b-bound sRNA (Right, each panel). 21-nt sRNAs were shown in (**a**) and (**c**), and 24-nt sRNAs were shown in (**b**) and (**d**). All sRNAs were shown in (**a-b**), while phasiRNAs were shown in (**c-d**). All the tassel samples were grown in 2015

Several *Arabidopsis* and rice AGOs have been shown to bind sRNA populations with a strong preference at their 5′-terminal nucleotides [39]. To uncover AGO18b binding preference to sub-populations of 21-nt sRNAs and phasiRNAs, we analyzed the base composition of different sRNA libraries in this study. There was no preference for the 5′-nucleotide in the unselected 21-nt and the 24-nt sRNAs (Fig. 3a-b, left panel**)**. The 5′- uridine (5′-U) preference sharply emerged in the AGO18b- associated 21-nt sRNAs, up to 62–66% of all AGO18b-bound 21-nt reads (Fig. 3a, right panel). The 5′-adenine (5′-A) preference in the AGO18b-bound 24-nt sRNA reached 53% of all AGO18b-bound 24-nt reads (Fig. 3b, right panel). AGO18b binding preference of 5′-U in the 21-nt sRNA population and of 5′-A in the 24-nt sRNA population was the same in both W22-ref and *ago18b::mum* samples, and in both years of sampling (Additional file 1: Figure S3a-b). These results indicate that AGO18b could selectively bind to both the 5′-U-containing 21-nt sRNAs and the 5′-A-containing 24-nt sRNAs, suggesting the sRNA length dependent 5′-nucleotide preference of AGO18b binding. Possible explanations are either that AGO18b has a binding capability of 5′-U or 5′-A, or alternatively, that AGO18b binding of 24-nt siRNAs may be caused by a partner AGO protein physically interacting with AGO18b.

We then analyzed the 5′-base composition of 21-nt and 24-nt phasiRNAs in sRNA-seq data. Neither type of phasiRNAs showed a 5′-nucleotide preference (Fig. 3c-d, left panel). However, the preference of the 21-nt 5′-U and the 24-nt 5′-A phasiRNAs was evident in the AGO18b-bound sRNAs (Fig. 3c-d and Additional file 1: Figure S3c-d, right panel), and AGO18b binding profile in *ago18b::mum* was quite similar to that in W22-ref (Fig. 3c-d, and Additional file 1: Figure S3c-d).

### AGO18b binds to miR2275 required for 24-nt siRNA/phasiRNA production

So far, we have shown that AGO18b may negatively regulate the expression of 24-nt phasiRNAs (Fig. 1c). However, we found that AGO18b binds preferentially to 21-nt phasiRNAs/sRNAs with a 5′-uridine and 24-nt phasiRNAs/sRNAs with a 5′-adenine (Fig. 3 and Additional file 1: Figure S3). We notice that most maize miRNAs contain a 5′-uridine (Additional file 1: Figure S4a). It is known that miR2275 and miR2118 are involved in the production of 24-nt and 21-nt phasiRNAs respectively [35], and both miRNAs contain a 5′-uridine. To further explore the possible mechanism of AGO18b preferentially binding of 21-nt over 24-nt phasiRNAs, we decided to analyze the expression and AGO18b-association of these two miRNAs.

**Fig. 4:**
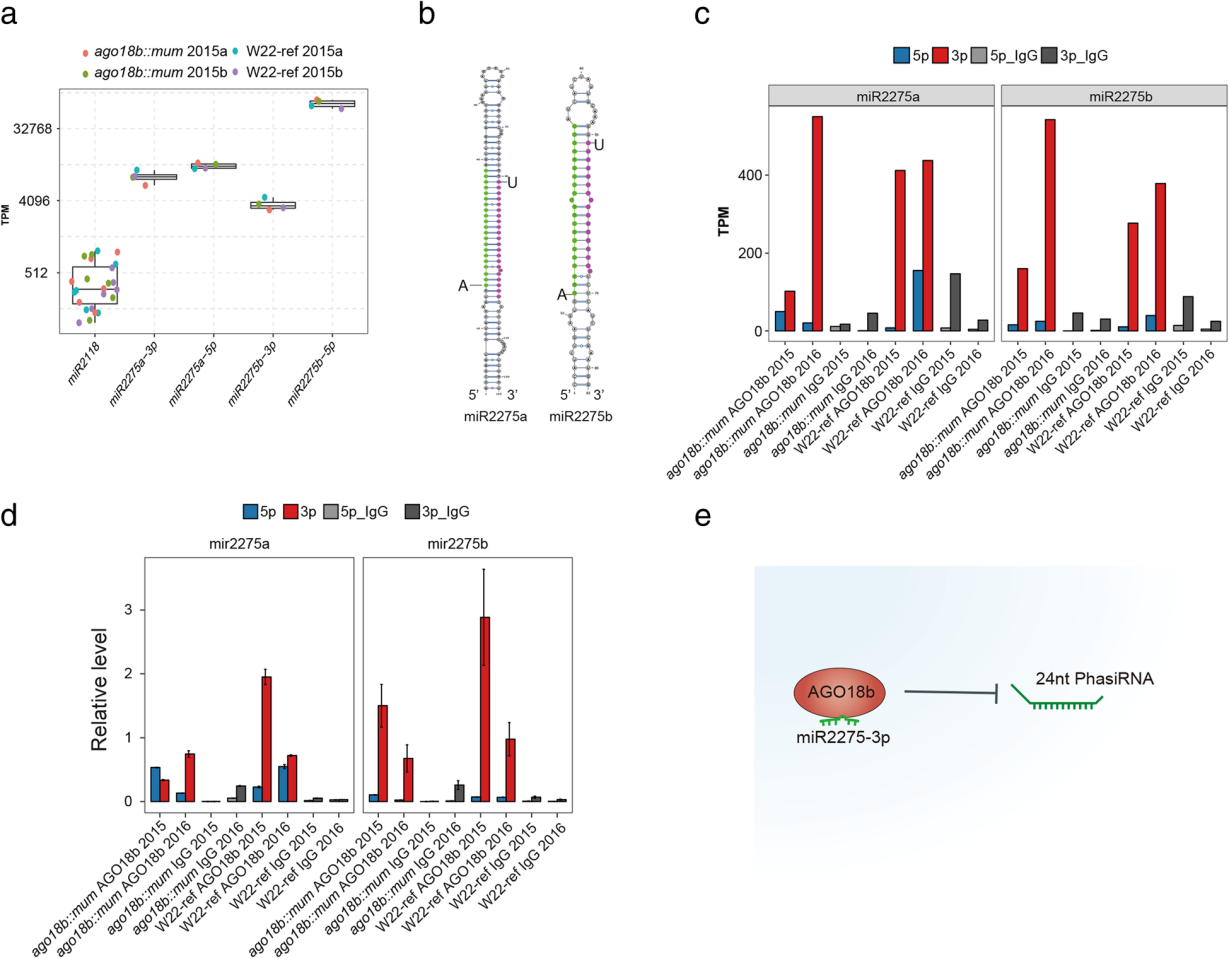
The AGO18b binding feature on miR2275 and its impact on 24-nt phasiRNA production. **a** Box plot showing the expression level of miR2118 and miR2275 for sRNA libraries, respectively. All miR2218 isoforms (miR2218a-miR2218g) were shown in one box together. **b** The secondary structure of zma-miR2275a and zma-miR2275b. Green and purple nucleotides represent the 5p and 3p strand of mature miRNA, respectively. **c** Bar plot showing the expression level for 5p and 3p miRNAs of miR2275a and miR2275b, respectively. AGO18b and IgG samples were plotted side by side. **d** Bar plot showing the relative level of miR2775a and miR2775b by RT-qPCR validation. **e** Model diagram showing the possible mechanism that AGO18b may sequester miR2275 3p and therefore prevents the 24-nt phasiRNA production

Interestingly, we found that the expression level of miR2275 reached two orders of magnitude higher than that of miR2118 in the premeiotic tassels (Fig. 4a). However, the amount of 24-nt siRNAs was only about 2–3 folds higher than that of 21-nt siRNAs (Additional file 1: Figure S1b), suggesting the presence of additional mechanisms in regulating the activity of these two miRNAs. We noticed that the 5p strand of miR2275 family members expressed at a higher level than the 3p strands (Fig. 4a). However, their 3p strand contains a 5′-U but the 5p strand contains a 5′-A (Fig. 4b), suggesting that 3p strand may be associated with AGO18b.

Consistent with this prediction, miR2275-3p strand was enriched by AGO18b while miR2275-5p strand was excluded (Fig. 4c and Additional file 1: Figure S4b). MiR2118 was also enriched when compared to the extremely low abundance in the IgG samples (Additional file 1: Figure S4b). The specific enrichment of AGO18b binding to miR2275-3p was also validated by quantitative PCR (Fig. 4d). Taken together the AGO18b binding of miR2275-3p and its negative regulation of the 24-nt phasiRNA production, we speculate that AGO18b sequesters miR2275-3p and therefore prevents the 24-nt phasiRNA generation **(**Fig. 4e).

### AGO18b binds to miR166a and other miRNAs

**Fig. 5:**
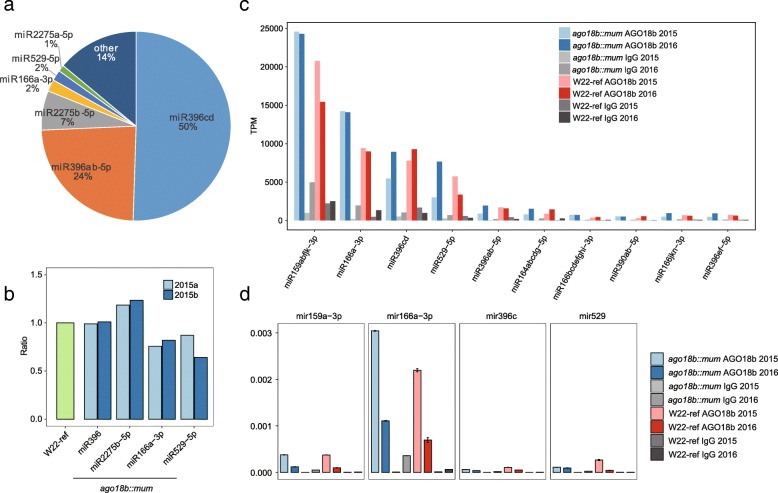
The expression regulation of miR166a by AGO18b binding. **a** Pie chart showing the percentage distribution for miRNAs in the total miRNA reads. **b** Bar plot showing the ratio of miRNA between W22 and *ago18b::mum* samples from sRNAome sequencing data. **c** Bar plot showing the abundance (TPM) of AGO18b-associated miRNAs in AGO18b-associated sRNA libraries. **d** Bar plot showing the relative abundance of AGO18b-associated miRNAs relative to IgG controls by quantitative PCR experiment

Previous study has shown that AGO18b functions in tassel development perhaps by interacting with the miR166-mediated regulatory pathway [31], but the binding ability to miR166 of AGO18b was not verified. AGO18b binding to 21-nt sRNAs/phasiRNAs with a 5′-U and 24-nt sRNAs/phasiRNAs with a 5′-A cannot explain its negative regulation of IM development. To uncover the role of AGO18b in maize IM development, we analyzed the abundance of all miRNAs, and found that four members of the miR396 family were the most abundant miRNAs in maize tassel, in total accounting for ~ 74% of the detected miRNAs (Fig. 5a and Additional file 1: Figure S4c). MiR2275b-5p was the next most abundant, followed by miR166-3p (Fig. 5a and Additional file 1: Figure S4c). A slight decrease in miR166a-3p and miR529-5p in *ago18b::mum* tassels was found by sRNA-seq data analysis (Fig. 5b). We then analyzed the AGO18b-bound miRNAs, and found that a significant enrichment in miR159abfjk-3p, miR166a-3p, miR529-5p by AGO18b in W22-ref and *ago18b::mum* tassels by cRIP-seq data (Fig. 5c) and RT-qPCR (Fig. 5d). Most of these AGO18b-enriched miRNAs showed a 5′-U preference although mature maize miRNAs don’t show obviously 5′-nucleotide preference (Additional file 1: Figure S4a). We noticed that the sequence of miR166-3p is exactly identical sequence as the *Arabidopsis* miR166 sequestered by AGO10. MiR166 is a known negative regulator of SAM development in *Arabidopsis* [40]. The lower level of miR166-3p may partially contribute to the longer tassel phenotype in *ago18b::mum*.

### AGO18b binds to the targets of miR166a for a negative regulation

**Fig. 6:**
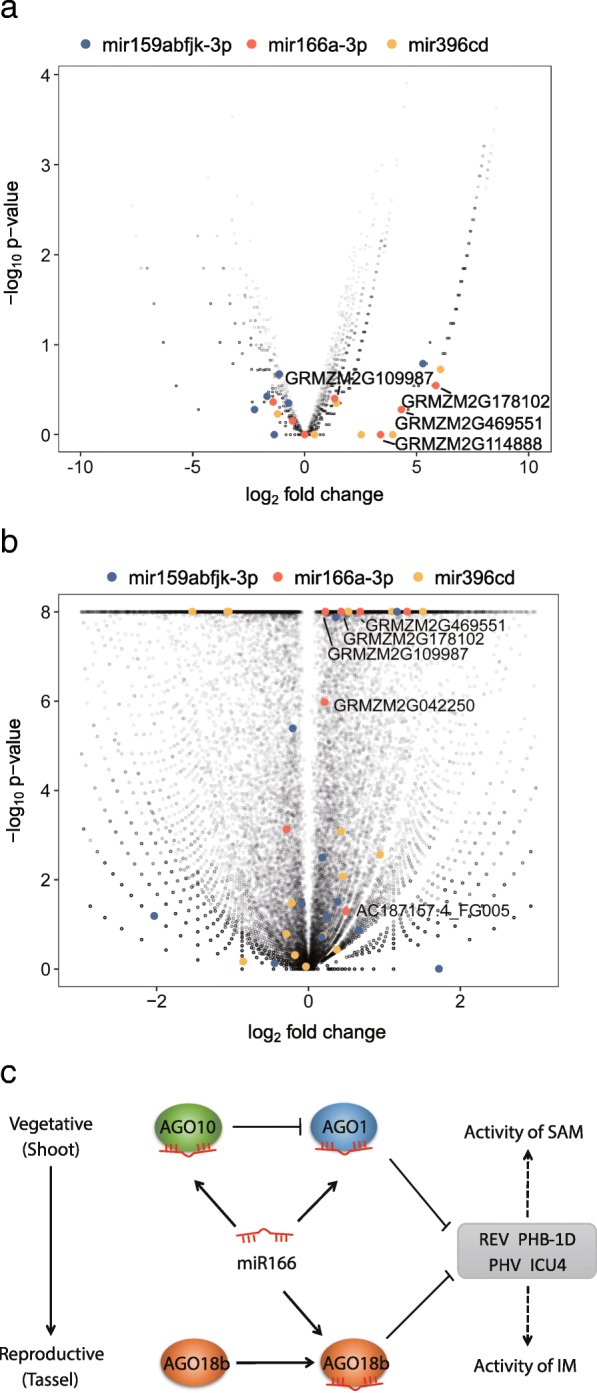
The AGO18b binding profile on the targets of miR166a for a negative regulation. **a** Volcano plot of the mRNAs specifically bound by AGO18b. The ratio of mRNA abundance in AGO18b RIP vs IgG RIP samples from *ago18b::mum* pre-meiotic tassels was normalized and plotted. Targets of mir159abfjk-3p, mir166a-3p and mir396cd were labeled with a different color, and targets of mir166a-3p were indicated with gene IDs. **b** Volcano plot of the AGO18b-regulated genes. The ratio of mRNA abundance in the RNA-seq samples from the *ago18b::mum* vs W22 pre-meiotic tassels was normalized and plotted. **c** A model to illustrate the AGO18b regulation of shoot apical meristem (SAM) and inflorescence meristem (IM) activity

The AGO18b binding ability to miR166a indicated the possibility that AGO18b regulates the SAM development by repressing the expression level of miR166a targets. To test this hypothesis, we analyzed the mRNA reads cross-linked with AGO18b (Additional file 2: Table S1). Large fraction (25–32%) of AGO18b-associated long mRNA/non-coding RNAs was mapped to the CDS region of the maize genome, which was higher than those of the sRNA-seq libraries (Additional file 1: Figure S5a). We then predicted the targets of the AGO18b-bound miR166a-3p and miR159a/b/f/j/k-3p, using the less-bound miR396c/d as control (Fig. 6a and Additional file 1: Figure S5b). A total of 11 maize genes were predicted to contain miR166a target sites. Of these, six were annotated as HD-ZIP III family TFs and the other five had no annotation (Additional file 2: Table S4). Many targets of miR166a-3p and miR159a/b/f/j/k-3p were enriched in the AGO18b-bound mRNAs, while fewer were enriched for those of miR396c (Fig. 6a). Transcripts from four maize genes that were homologous to the HD-ZIP III family TFs *PHV*, *PHB-1D*, *REV* and *ICU/ATHB-5* were significantly bound by AGO18b (Fig. 6a, Additional file 2: Table S4). Our previous study have shown that most mRNAs associated by AGO18b-miR166a were up-regulated in the *ago18b::mum* tassels [31], indicating that the AGO18b-miRNA-mRNA complex is functional (Fig. 6b). In conclusion, AGO18b strongly and directly associates with miR166a-3p and its mRNA targets, which agrees well with the negative regulatory phenomenon of AGO18b. These results together suggest that, similar to AGO1, AGO18b can bind to HD-ZIP III family TFs and inhibit the IM activity during the tassel development via miR166 pathway (Fig. 6c).

## Discussion

For the inflorescence architecture of *Arabidopsis,* previous studies have shown that AGO1 is required for the maintenance of inflorescence meristem through regulating *TERMINAL FLOWER1* expression [41, 42]. Although there is an obvious morphological difference between maize and *Arabidopsis* inflorescence, recent evidence shows that maize AGO1a can nearly restore the *Arabidopsis ago1–27* to wild-type phenotype [43], suggesting the functional conservation of AGO1 in maize and *Arabidopsis*. In our previous study, we found that repressed AGO18b resulted in increased plant height and elongated length of tassel main inflorescence in maize [31]. The increased spikelet number in reduced AGO18b maize plants is highly similar to that of AGO1 in *Arabidopsis,* suggesting that maize AGO18b functions as a negative regulator.

In *Arabidopsis*, the HD-ZIP III genes are regulated by miR165/166 [40, 44, 45]. In this study, we found that maize AGO18b binds the miR166-3p. We also detected the direct association between AGO18b and the transcripts of HD-ZIP III TF-encoding genes (GRMZM2G109987, GRMZM2G042250, GRMZM2G469551, and GRMZM2G178102), which were putative targets of zma-miR166a (Fig. 6a, and Additional file 2: Table S4). The results provide the direct evidence that AGO18b leads to the repression or cleavage of the miR166a-guided mRNA targets. Therefore, maize AGO18b is validated to be involved in the regulation of maintenance of inflorescence meristem by binding to miR166a and HD-ZIP III TF regulatory factors in maize.

In addition to AGO1, AGO10 is also involved in the regulation of the floral organ identity and floral determinacy in *Arabidopsis* [18] and the maintenance of SAM in rice [46] by binding competitively to miR165/166 [11, 15]. Phylogenetic analysis shows that maize AGO1/10/18 are grouped into the *Arabidopsis* AGO1/5/10 clade [8], indicating that maize AGO1/10/18 perform similar biological functions in maize as their orthologs in *Arabidopsis*. In maize, members of the AGO1 family were expressed at high levels in the early stage of tassel development, then gradually decreased their expression accompanied by a gradual increase of AGO18b with the development of tassel [31] . The opposite expression patterns of maize AGO18b and AGO1 suggest that these AGO proteins are restricted from expressing at a certain developmental stage. As shown in Fig. 6c, AGO18b increases expression level gradually with tassel development, and accumulates to a high level in the meiotic anther. AGO1 is proposed to play a major role in repressing the activity of inflorescence meristem. AGO10 selectively sequesters miR166 to alleviate repression of AGO1 as in *Arabidopsis* and rice. When AGO18b is gradually enriched in tassel meristem, the AGO18b-miR165/166-HD ZIP III regulatory pathway is enhanced to repress the maintenance of inflorescence meristem by coordinating with AGO1 (Fig. 6c). Thus, the fine spatiotemporal accumulation of maize AGO1, AGO10, and AGO18b highlights the importance of AGO18b in the regulation of tassel development, and also implies that a multiplex regulatory system consisting of AGO1, AGO10, and AGO18b functions in maize tassel to fine-tune inflorescence meristem activity.

Investigations in animal and plant species have demonstrated that gametogenesis and germline development requires involvement of AGO proteins and specific sRNAs, such as Piwi protein and 26~32-nt piRNAs in *Drosophila* [47], AGO9 and TE-derived sRNAs in *Arabidopsis* [48], AGO9/AGO104 and sRNA-mediated non-CG methylation in maize [49], AGO5c/MEL1 and 21-nt phasiRNAs in rice [37]. As mentioned above, maize AGO18b specifically regulates maintenance of inflorescence meristem via miR166a-HD-ZIP III TF regulatory pathway to differentiate or terminate spikelets or flowers in an orderly way. However, microRNAs constitute a very small fraction of the sRNA population (Fig. 1b). Zhai et al. (2015) have found that 21-nt phasiRNAs are predominant while 24-nt phasiRNAs occupy a small fraction of sRNA population in premeiotic tassel [38]. The expression profile of AGO18b matches the expression timing of meiotic phasiRNAs, suggesting that AGO18b is the partner of the meiotic phasiRNAs [38]. In this study, we found that 21-nt phasiRNAs in premeiotic tassel greatly resemble those in anther but 24-nt phasiRNAs were different from those in anther, suggesting that 24-nt phasiRNAs mainly play a role in other floral tissues but not anthers. Because 24-nt phasiRNAs are transcribed in other floral organs besides anthers, characteristics of AGO binding to sRNAs and tissue-specific distribution of 24-nt phasiRNAs indirectly suggests that AGO18b functions in the regulation of inflorescence meristem development by binding to 24-nt phasiRNAs.

Some AGOs tends to bind sRNAs with a strong preference for their 5′-terminal nucleotides in *Arabidopsis* [39] and rice [37, 50]. Interestingly, we found that maize AGO18b varies its affinity to sRNAs with two different 5′-nucleotides in a length dependent manner. AGO18b strongly selects 21-nt phasiRNAs with a 5′-U, and 24-nt phasiRNAs with a 5′-A. Selection of 21-nt sRNA with a 5′-U is also evident by its bound miRNAs (Fig. 5). It is unclear whether AGO18b association of 24-nt phasiRNAs would be enhanced by an associated secondary factor during meiosis, during which 24-nt phasiRNAs is predominantly expressed [38]. An alternative model is that AGO18b strongly associates with 21-nt phasiRNAs but much more weakly associates with 24-nt phasiRNAs, and its preferred association with 21-nt phasiRNAs allows a very low fraction of this class of phasiRNAs to function well in maize tassel. The flexible selection by AGO18b could expand its regulatory spectrum, although the mechanism of the flexible selection and the function of AGO18b-phasiRNAs association are still unclear.

## Conclusions

In this study, we performed a modified RIP-seq method to capture the bound sRNAs and mRNAs by maize AGO18b. Our data revealed the specific binding features of AGO18b is essential to its functions in tassel development. These results suggest that coordinating tassel development is controlled by AGO18b binding of miRNAs and/or phasiRNAs. The regulatory mechanism may be also applied to other inflorescence-related AGOs, and could be potentially used for plant breeding. Meanwhile, the influence of the interaction between AGO18b and phasiRNAs need to be further investigated in future studies.

## Methods

### Plant materials

A Mutator-mediated Uniform mutant (UFMu01249) at AGO18b was provided by the Maize Stock Center (http://archive.maizegdb.org/stock.php). To reduce the impact of background mutations, the Mu-inserted heterozygotes were crossed with W22, a background line for UFMu01249, followed by 2 cycles of backcrossing to W22. The identification of Mu-inserted individuals and individuals growth condition were the same as described in the study of Sun et al. [31], so we didn’t fully describe the materials in this study. The experimental research in study were conducted in compliance with Experimental Plants Regulations from the Chinese Ministry of Science and Technology.

### Reverse transcription quantitative PCR (RT-qPCR)

Total RNAs were extracted using the TRIzol Reagent (Life Technologies, Invitrogen). After removing genomic DNA contamination by DNase I (TaKaRa Biotech, Dalian, China), M-MLV reverse transcriptase (Life Technologies, Invitrogen) was used to synthesize cDNA. The SYBR Green RT-qPCR kit (Bio-Rad, Hercules CA, USA) was used for RT-qPCR experiment with gene-specific primers (Additional file 3: Table S5) with three biological replicates. For microRNA expression analysis, 18S gene was used as the internal control (Additional file 3: Table S5).

### Cross-linking RNA immunoprecipitation (cRIP) of AGO18b-bound RNAs

By performing alignment analysis, we found the first 13 amino acids of AGO18b were different from that of AGO18a, and then used as antigens for antibody preparation [8]. The premeiotic tassels (~ 6-cm) were collected from *ago18b::mum* and W22. As far as we know the plant cells encase themselves within a firm and complex polysaccharide wall [51], which may resist UV penetrate and therefor reduce crosslinking efficiency. A higher total energy can be employed for tissues in suspension, and multiple rounds of UV exposure with intermittent mixing are needed in order to obtain evenly distributed crosslinking throughout the sample [52]. Therefore, we irradiated twice at 800 mJ/cm^2^ for obtain higher crosslinking efficiency and evenly distributed crosslinking throughout the sample. One gram of frozen tissues were sliced and resuspended in 1 V/w of PBS in petri dishes, then placed dishes on ice during irradiated twice at 800 mJ/cm^2^. After cross-linking, the tissues were pulverized in liquid nitrogen and were lysed in ice-cold lysis buffer (50 mM Tris 7.4, 150 mM NaCl, 2 mM EDTA, 0.1% SDS, 0.5% NP-40, and 0.5% deoxycholate) with freshly added 1 mM DTT, 200 U/mL RNase inhibitor (Takara) and protease inhibitor cocktail (Roche) for 30 min on ice. After centrifugation for 30 min at 12,000 rpm at 4 °C, the supernatant was transferred to a new tube and used for immunoprecipitation. For immunoprecipitation, 300 μL lysate was incubated with 10 μg self-raised rabbit polyclonal antibody against AGO18b or control IgG antibody overnight at 4 °C. The immunoprecipitates were further incubated with protein A Dynabeads for 2 h at 4 °C. After magnetic separation and removal of the supernatants, the beads were sequentially washed with lysis buffer, high-salt buffer (250 mM Tris 7.4, 750 mM NaCl, 10 mM EDTA, 0.1% SDS, 0.5% NP-40, and 0.5 deoxycholate), and PNK buffer (50 mM Tris, 20 mM EGTA and 0.5% NP-40) twice. The immunoprecipitates were eluted from the beads with buffer (50 nM Tris 8.0, 10 mM EDTA, and 1% SDS) and the RNA was purified with Trizol reagent (Life Technologies, Invitrogen, Carlsbad, CA, USA).

### cRIP-seq small RNA library preparation and analysis

The small RNAs in the recovered AGO18b-bound or IgG-bound RNA pools were amplified into sequencing libraries through a small RNA library preparation protocol following the manufacturer’s instruction (GnomeGen, Suzhou). The recovered libraries were sequenced on the illumina NextSeq 500 platform to generate 151-nt paired-end reads (ABLife Inc., Wuhan, China). The base-paired reads were then treated as single reads.

Raw reads were discarded if they contained N bases, and then were processed by clipping the adaptor and removing low quality bases (less than 20). Reads that were too short (less than 16-nt) were also removed. FASTX-Toolkit (Version 0.0.13) was used to recover the quality filtered reads. Filtered reads were then aligned to the reference genome (B73 RefGen_v3) by TopHat2 [53] with no more than 2 mismatches. The alignment result files were used to perform phasiRNA analysis. The reads were also aligned to the maize mature miRNAs from miRBase (version 21) by bowtie [54] with 1 mismatch. TPM value (Tag Per Million) was calculated for each miRNA. The sRNA TPM values of both *ago18b::mum* and W22 samples were treated as the binding density of AGO18b and were compared with the IgG samples directly. The binding density between *ago18b::mum* and W22 samples were also compared. Base composition analysis was performed by WebLogo software (http://weblogo.berkeley.edu/logo.cgi).

### cRIP-seq mRNA library preparation and analysis

A portion of RNAs AGO18b-bound or IgG-bound RNAs was used to construct libraries using an mRNA library preparation kit (GnomeGen, Suzhou) according to the manufacture’s instruction. The recovered libraries were then applied to illumina NextSeq 500 platform for 151-nt pair-end sequencing (ABLife Inc., Wuhan, China). Raw reads filtration and alignment were the same way as for sRNA RIP-seq libraries. Aligned reads with more than one genomic locations were discarded to avoid ambiguity. AGO18b-bound peaks were called using the followed strategy. After alignment, reads with same genome location were counted and merged as unique reads. The aligned clusters were obtained by merging overlapped unique reads according to their genome coordinates. The “*in silico* random IP” strategy [10] was used to select the high-confidence binding sites compared with the IgG background. This process was repeated for 500 times. False discovery rate (FDR) was determined by counting the observed number of maximum clusters from each of 500 repeats. Peaks with FDR ≤ 0.01 were treated as AGO18b-bound sites. Genes with at least one AGO18b-bound peaks were treated as AGO18b-bound genes. This strategy was performed on both *W22-ref* and *ago18b::mum* samples.

### Small RNAome analysis

The small RNAome sequencing data (sRNA-seq) was obtained from Sun et al. [31]. Raw reads filtration and the following alignment were the same as the RIP-seq small RNA libraries. For each miRNA, its target mRNA was predicted using web software psRNATarget [55] with default parameters. The input files were the mature miRNA sequence and the mature mRNA sequence (including CDS and UTR) without poly (A) tail. The output file contains the miRNA-target pairs with statistical information.

### PhasiRNA analysis

To analyze the feature of AGO18b-bound phasiRNAs, the genomic coordinates of recently identified maize phasiRNAs [38] were used as the input. The RIP-seq and sRNA-seq reads were then aligned to the genomic coordinates of 21-nt and 24-nt phasiRNA loci. The reads per kilobase per million (RPKM) value of each phasiRNA was calculated for AGO18b binding density (cRIP-seq) or expression level (sRNA-seq).

## Additional files

Additional file 1: **Figure S1.** The composition of sRNA libraries of pre-meiotic maize tassels. **Figure S2.** RIP method application and the phasiRNA expression level. **Figure S3.** AGO18b binds to 21-nt and 24-nt sRNAs/phasiRNAs with base preference. **Figure S4.** AGO18b-associated miRNA abundance. **Figure S5.** AGO18b association of mRNAs and the related functions. (DOCX 1364 kb)

Additional file 2: **Table S1.** Sequencing and alignment information of the Illumina-sequenced samples in this study. **Table S2.** Sequencing reads length distribution of small RNA libraries for wild-type and *ago18b::mum* samples, respectively. **Table S3.** The mapping result of sRNA libraries to the phasiRNAs loci. **Table S4.** The expression level of miR166a targets in RNA-seq and mRNA RIP-seq libraries. (DOCX 23 kb)

Additional file 3: **Table S5.** List of primer sequences used in the study. (XLS 30 kb)

## Data Availability

Sequence data from this article were deposited at the Gene Expression Omnibus (http://www.ncbi.nlm.nih.gov/geo/) under accession number GSE93983.

## Abbreviations

AGO: Argonaute
IM: Inflorescence meristem
phasiRNAs: Phase associated siRNAs
RIP: RNA immunoprecipitation
SAM: Shoot apical meristem
TF: Transcription factors
