# Supplementary material for: Genome-wide identification of AGO18b-bound miRNAs and phasiRNAs in maize by cRIP-seq
Source: BMC Genomics. 2019 Aug 16;20:656. doi: 10.1186/s12864-019-6028-z (PMC6697968; doi:10.1186/s12864-019-6028-z)
Supplement: Supplementary file 1 — Figure S1. The composition of sRNA libraries of pre-meiotic maize tassels. Figure S2. RIP method application and the phasiRNA expression level. Figure S3. AGO18b binds to 21-nt and 24-nt sRNAs/phasiRNAs with base preference. Figure S4. AGO18b-associated miRNA abundance. Figure S5. AGO18b association of mRNAs and the related functions. (DOCX 1364 kb) [file 12864_2019_6028_MOESM1_ESM.docx]

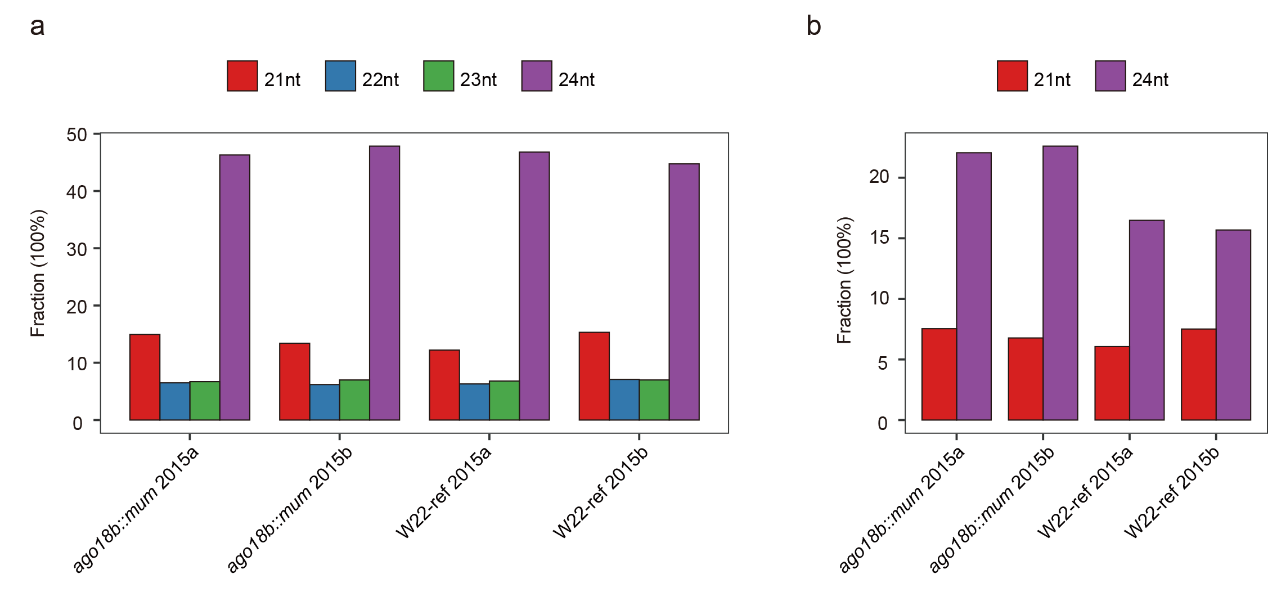


**Fig. S1.** **The composition of sRNA libraries of premeiotic maize tassels.** (**a**) Bar plot of the percentage for 21-24nt sRNA reads in sRNA libraries. (**b**) Bar plot of the percentage for 21nt and 24nt phasiRNAs in sRNA libraries.


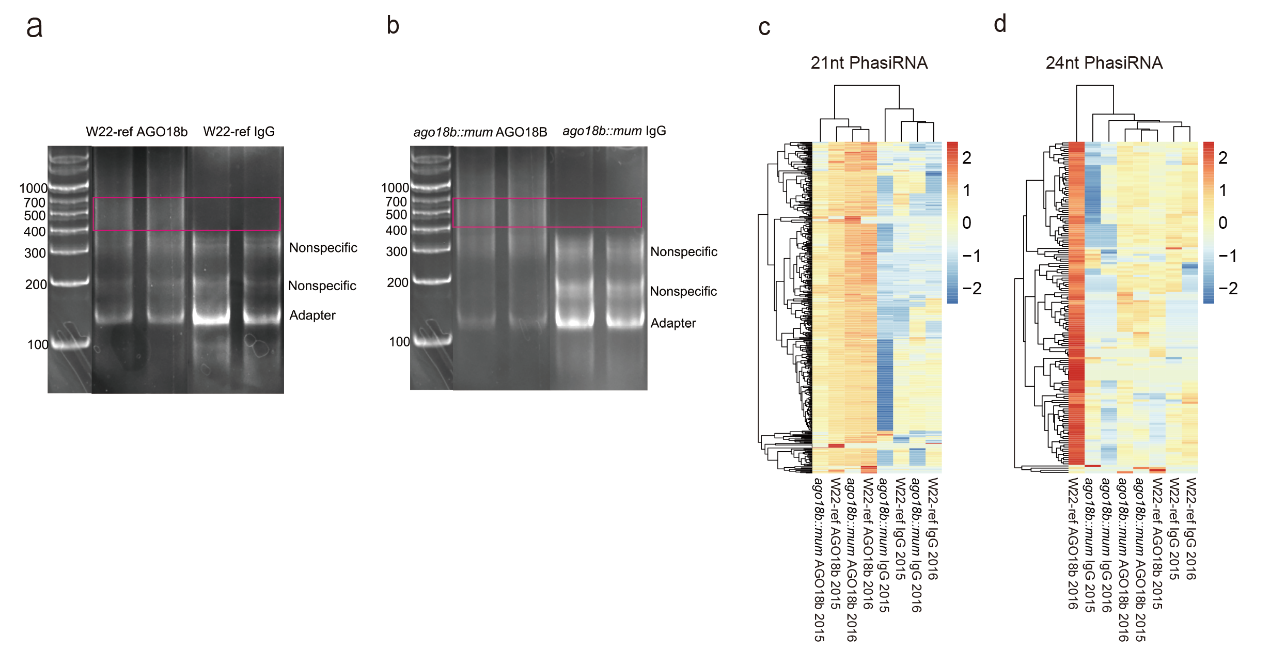


**Fig. S2.** **RIP method application and the phasiRNA expression level.** (**a-b**) The analysis of Nu-PAGE separated protein-RNA complexes for W22-ref (a) and *ago18b::mum* samples (b), respectively. Pink rectangle blocks were the regions selected for the following library construction. The cDNA populations showed the significant difference between AGO18b antibody and IgG samples in their intensities on a gel. (**c**) Hierarchical clustering heatmap of 21nt phasiRNA expression level in AGO18b-bound and IgG control samples. (**d**) Hierarchical clustering heatmap of 24nt phasiRNA expression level in AGO18b-bound and IgG control samples.


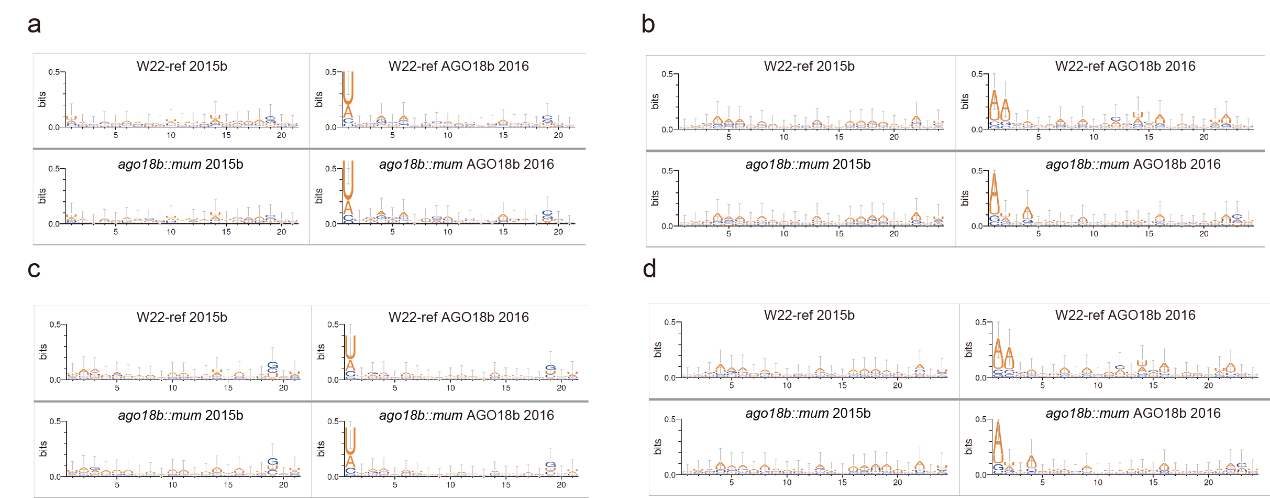


**Fig. S3.** **AGO18b binds to 21-nt and 24-nt sRNAs/phasiRNAs with base preference.** Base composition of transcriptome sRNA (Left, each panel) and AGO18b-bound sRNA (Right, each panel). 21-nt sRNAs are shown in **a** and **c**, and 24-nt sRNAs are in **b** and **d**. All sRNAs are shown in **a-b**, while phasiRNAs are in **c-d**. The tassel samples for transcriptome sRNA were grown in 2015, and those for AGO18b immunoprecipotation were grown in 2016.


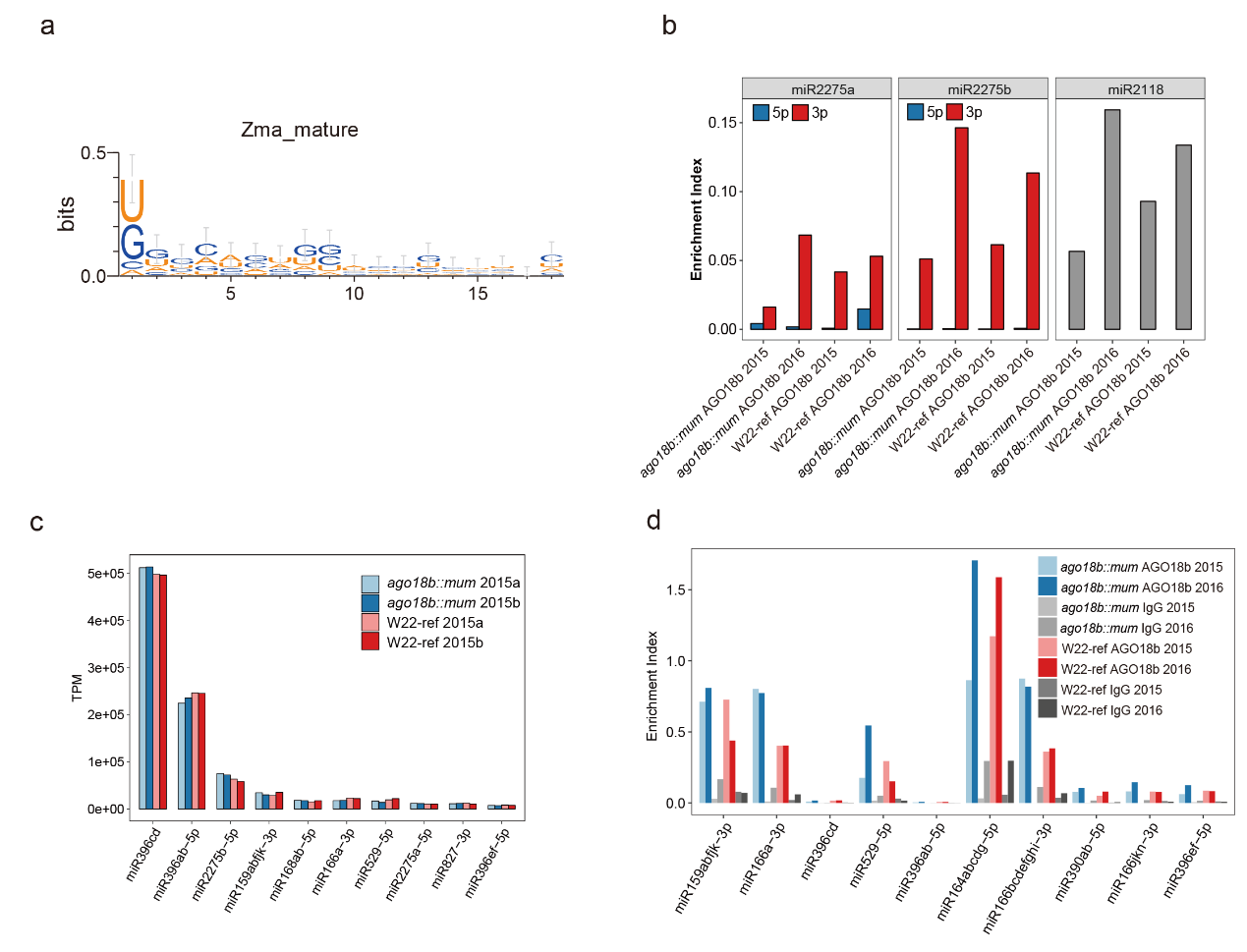


**Fig. S4.** **AGO18b-associated miRNA abundance.** (**a**) Base composition of the maize mature miRNAs showed the preference of 5’ uridine. (**b**) Bar plot of the enrichment index for 5p and 3p miRNAs of miR2275a and miR2275b, respectively. MiR2118 was plotted alone due to the indiscriminate of 5p and 3p. (**c**) Bar plot of the expression level for miRNAs by TPM value in sRNA libraries. MiR396 family was the most abundant. (**d**) Bar plot of the miRNA enrichment index in AGO18b-associated samples. AGO18b and IgG samples were plotted side by side.


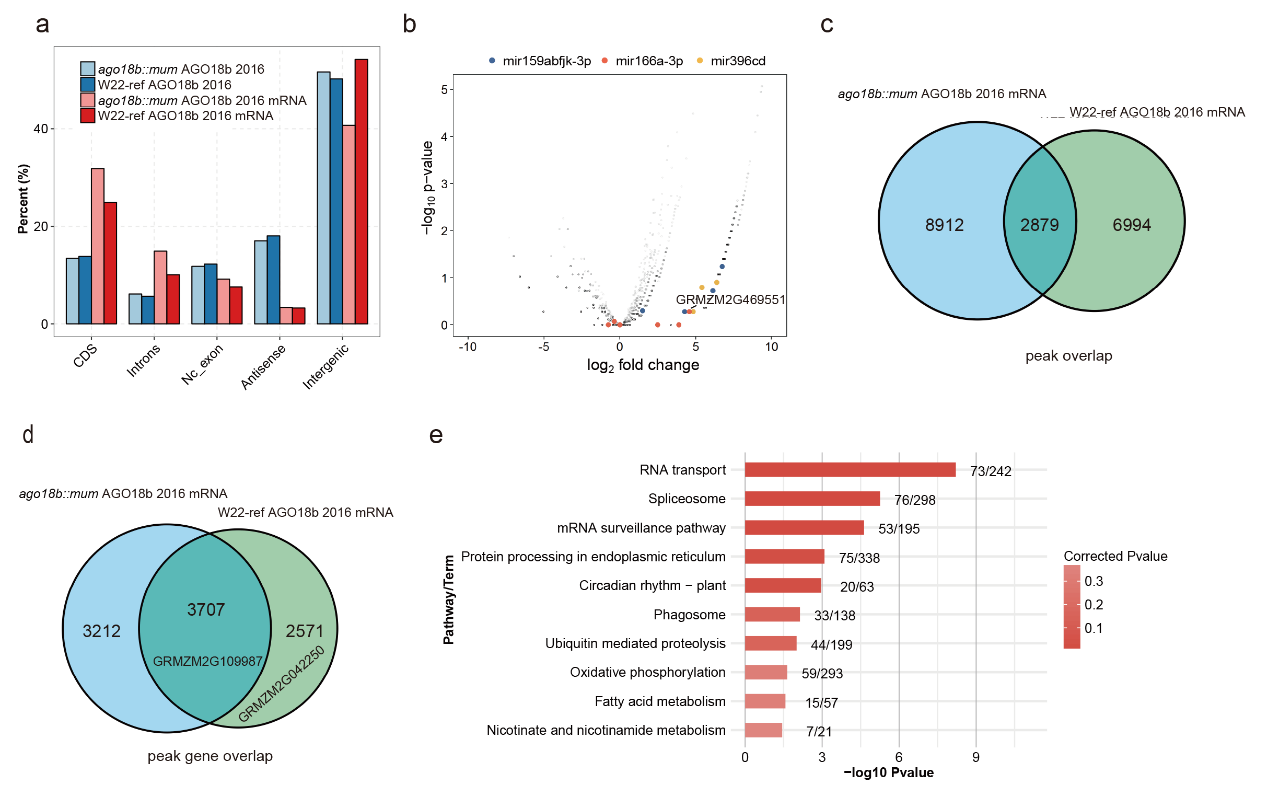


**Fig. S5.** **AGO18b association of mRNAs and the related functions.** (**a**) Bar plot of the reads region distribution for sRNA and mRNA RIP-seq in W22 and *ago18b::mum* samples, respectively. (**b**) Volcano plot of the gene abundance for AGO18b RIP vs IgG RIP in W22-ref. Targets of mir159abfjk-3p, mir166a-3p and mir396cd were labeled with different color, and targets of mir166a-3p were annotated with gene ID. (**c**) Venn diagram for the peaks overlap from the *ago18b::mum* AGO18b RIP and W22-ref AGO18b RIP in 2016. (**d**) Venn diagram for the genes overlap from the *ago18b::mum* AGO18b RIP and W22-ref AGO18b RIP in 2016. Targets of miR166a-3p were labeled in the figure. (**e**) Bar plot of the enriched KEGG pathways for genes belonging to overlapped peak in (c).
