# Supplementary material for: Genome-wide identification of AGO18b-bound miRNAs and phasiRNAs in maize by cRIP-seq
Source: BMC Genomics. 2019 Aug 16;20:656. doi: 10.1186/s12864-019-6028-z (PMC6697968; doi:10.1186/s12864-019-6028-z)
Supplement: Supplementary file 2 — Table S1. Sequencing and alignment information of the Illumina-sequenced samples in this study. Table S2. Sequencing reads length distribution of small RNA libraries for wild-type and ago18b::mum samples, respectively. Table S3. The mapping result of sRNA libraries to the phasiRNAs loci. Table S4. The expression level of miR166a targets in RNA-seq and mRNA RIP-seq libraries. (DOCX 23 kb) [file 12864_2019_6028_MOESM2_ESM.docx]

**Table S1:** Sequencing and alignment information of the Illumina-sequenced samples in this study.

| Sample | Type | Raw | Clean | Unique reads | Total aligned | Unique aligned | Multiple aligned |
| --- | --- | --- | --- | --- | --- | --- | --- |
| WT AGO18b 2015 | sRNA cRIP-seq | 41830914 | 23495140(56.17%) | 7837021(33.36%) | 20177212(85.88%) | 5151720(25.53%) | 15025492(74.47%) |
| WT IgG 2015 |  | 6365816 | 1428815(22.45%) | 578070(40.46%) | 991688(69.41%) | 160845(16.22%) | 830843(83.78%) |
| MuT AGO18b 2015 |  | 10262710 | 6373353(62.10%) | 2307325(36.20%) | 5516527(86.56%) | 1170000(21.21%) | 4346527(78.79%) |
| MuT IgG 2015 |  | 1310134 | 173404(13.24%) | 87605(50.52%) | 74320(42.86%) | 15133(20.36%) | 59187(79.64%) |
| WT AGO18b 2016 |  | 19875176 | 7266822(36.56%) | 1624390(22.35%) | 6053163(83.3%) | 2152106(35.55%) | 3901057(64.45%) |
| WT IgG 2016 |  | 13023146 | 1727397(13.26%) | 515334(29.83%) | 810507(52.28%) | 200954(24.79%) | 609553(75.21%) |
| MuT AGO18b 2016 |  | 21832066 | 6489283(29.72%) | 1634565(25.19%) | 5245783(80.84%) | 1630895(31.09%) | 3614888(68.91%) |
| MuT IgG 2016 |  | 11730060 | 1471981(12.55%) | 671436(45.61%) | 4289198(84.54%) | 866599(20.2%) | 3422599(79.8%) |
| WT AGO18b | mRNA  cRIP-seq | 39055714 | 33467880(85.69%) | 13438489(40.15%) | 20394523(60.94%) | 2347470(11.51%) | 18047053(88.49%) |
| WT IgG |  | 3653182 | 2857240(78.21%) | 1380399(48.31%) | 1479480(51.78%) | 150224(10.15%) | 1329256(89.85%) |
| MuT AGO18b |  | 31753498 | 25401342(80.00%) | 11272605(44.38%) | 15234664(59.98%) | 1891366(12.41%) | 13343298(87.59%) |
| MuT IgG |  | 9754390 | 8406406(86.18%) | 3802552(45.23%) | 4755653(56.57%) | 463809(9.75%) | 4291844(90.25%) |
| WT 2015a | sRNA-seq | 4843632 | 4381464(90.46%) | 1706931(38.96%) | 3748785(85.56%) | 1514885(34.57%) | 2233900(50.99%) |
| WT 2015b |  | 4811228 | 4046929(84.11%) | 1569903(38.79%) | 3552430(87.78%) | 1413183(34.92%) | 2139247(52.86%) |
| MuT 2015a |  | 6534428 | 5821636(89.09%) | 2045202(35.13%) | 5104164(87.68%) | 2160763(37.12%) | 2943401(50.56%) |
| MuT 2015b |  | 6806734 | 6124999(89.98%) | 2197742(35.88%) | 5302417(86.57%) | 2278269(37.20%) | 3024148(49.37%) |
| WT mRNA | mRNA-seq | 102167842 | 85230610(83.42%) | 34468293(40.44%) | 67392051(79.07%) | 54082217(80.25%) | 13309834(19.75%) |
| MuT mRNA |  | 84272972 | 68031279(80.73%) | 32094089(47.18%) | 52962095(77.85%) | 42843913(80.9%) | 10118182(19.1%) |

WT: W22-ref.

MuT: *ago18b::mum*.

**Table S2.** Sequencing reads length distribution of small RNA libraries for W22-ref (WT) and *ago18b::mum* (MU) samples, respectively. 13-40nt reads were presented in the table.

| **Length** | **MU_1** | **MU_1 %** | **MU_2** | **MU_2 %** | **WT_1** | **WT_1 %** | **WT_2** | **WT_2 %** |
| --- | --- | --- | --- | --- | --- | --- | --- | --- |
| 13 | 70594 | 1.21% | 47040 | 0.77% | 24622 | 0.56% | 70413 | 1.74% |
| 14 | 74383 | 1.28% | 48362 | 0.79% | 25381 | 0.58% | 66408 | 1.64% |
| 15 | 80339 | 1.38% | 53106 | 0.87% | 28680 | 0.65% | 66385 | 1.64% |
| 16 | 94111 | 1.62% | 65459 | 1.07% | 35708 | 0.81% | 74794 | 1.85% |
| 17 | 81056 | 1.39% | 58503 | 0.96% | 33814 | 0.77% | 61536 | 1.52% |
| 18 | 84862 | 1.46% | 65817 | 1.07% | 39582 | 0.90% | 63422 | 1.57% |
| 19 | 85093 | 1.46% | 72551 | 1.18% | 44200 | 1.01% | 61555 | 1.52% |
| 20 | 132439 | 2.27% | 121086 | 1.98% | 77628 | 1.77% | 94023 | 2.33% |
| **21** | **870594** | **14.95%** | **821253** | **13.41%** | **535055** | **12.21%** | **619684** | **15.33%** |
| 22 | 378520 | 6.50% | 379753 | 6.20% | 276241 | 6.30% | 287254 | 7.11% |
| 23 | 390805 | 6.71% | 428518 | 7.00% | 297976 | 6.80% | 283239 | 7.01% |
| **24** | **2694882** | **46.29%** | **2930238** | **47.84%** | **2049970** | **46.79%** | **1810464** | **44.78%** |
| 25 | 122132 | 2.10% | 139571 | 2.28% | 114159 | 2.61% | 95011 | 2.35% |
| 26 | 46529 | 0.80% | 53821 | 0.88% | 43629 | 1.00% | 34072 | 0.84% |
| 27 | 31377 | 0.54% | 39297 | 0.64% | 29273 | 0.67% | 21733 | 0.54% |
| 28 | 29284 | 0.50% | 36337 | 0.59% | 28109 | 0.64% | 19478 | 0.48% |
| 29 | 35691 | 0.61% | 45421 | 0.74% | 35691 | 0.81% | 23696 | 0.59% |
| 30 | 54867 | 0.94% | 67747 | 1.11% | 62639 | 1.43% | 38106 | 0.94% |
| 31 | 83718 | 1.44% | 101824 | 1.66% | 96657 | 2.21% | 56073 | 1.39% |
| 32 | 84620 | 1.45% | 109203 | 1.78% | 97428 | 2.22% | 55001 | 1.36% |
| 33 | 55469 | 0.95% | 71846 | 1.17% | 59008 | 1.35% | 31627 | 0.78% |
| 34 | 47423 | 0.81% | 62277 | 1.02% | 53140 | 1.21% | 26346 | 0.65% |
| 35 | 30403 | 0.52% | 42487 | 0.69% | 36814 | 0.84% | 16513 | 0.41% |
| 36 | 26377 | 0.45% | 36829 | 0.60% | 33369 | 0.76% | 13376 | 0.33% |
| 37 | 19999 | 0.34% | 29851 | 0.49% | 25064 | 0.57% | 9226 | 0.23% |
| 38 | 17656 | 0.30% | 26789 | 0.44% | 22230 | 0.51% | 7632 | 0.19% |
| 39 | 12641 | 0.22% | 19171 | 0.31% | 16740 | 0.38% | 4985 | 0.12% |
| 40 | 10698 | 0.18% | 16565 | 0.27% | 15404 | 0.35% | 4081 | 0.10% |

**Table S3.** The mapping result of sRNA libraries to the phasiRNAs loci.

| Samples | Input | Total Mapped | 21nt PAHS | 24nt PAHS |
| --- | --- | --- | --- | --- |
| *Ago18b::mum* 2015a | 5821636 | 5104164(87.68%) | 492221(8.46%) | 1426827(24.51%) |
| *Ago18b::mum* 2015b | 6124999 | 5302417(86.57%) | 467644(7.64%) | 1542121(25.18%) |
| W22-ref 2015a | 4381464 | 3748785(85.56%) | 298574(6.81%) | 794064(18.12%) |
| W22-ref 2015b | 4046929 | 3552430(87.78%) | 340882(8.42%) | 703555(17.38%) |

**Table S4.** The expression level of miR166a targets in RNA-seq and mRNA RIP-seq libraries.

| Gene | MuT | WT | MuT_Ago18b  _mRNA | MuT_IgG  _mRNA | WT_Ago18b  _mRNA | WT_IgG  _mRNA | TAIR_ID | Symbol | Description |
| --- | --- | --- | --- | --- | --- | --- | --- | --- | --- |
| AC187157.4_FG005 | 0.62 | 0.38 | 0 | 0 | 0 | 0 | AT1G52150 | ICU4 | INCURVATA 4 |
| GRMZM2G003509 | 2.44 | 2.62 | 4 | 2 | 3 | 0 | AT2G34710 | PHB-1D | PHABULOSA 1D |
| GRMZM2G023291 | 7.89 | 3.08 | 17 | 0 | 10 | 0 | AT1G30490 | PHV | PHAVOLUTA |
| GRMZM2G042250 | 18.97 | 14.34 | 15 | 4 | 4 | 1 | AT5G60690 | REV | REVOLUTA |
| GRMZM2G048297 | 0.41 | 0.33 | 0 | 0 | 0 | 0 | AT1G52150 | ICU4 | INCURVATA 4 |
| GRMZM2G109987 | 56.36 | 42.31 | 28 | 2 | 16 | 3 | AT5G60690 | REV | REVOLUTA |
| GRMZM2G114888 | 65.16 | 42.36 | 2 | 0 | 1 | 0 | AT1G64880 | -- | -- |
| GRMZM2G123644 | 0.02 | 0 | 0 | 0 | 0 | 0 | AT1G68580 | -- | -- |
| GRMZM2G178102 | 4.84 | 2.64 | 12 | 0 | 3 | 0 | AT2G34710 | PHB-1D | PHABULOSA 1D |
| GRMZM2G336718 | 0.01 | 0 | 0 | 0 | 0 | 0 | AT1G68580 | -- | -- |
| GRMZM2G423337 | 16.9 | 10.07 | 32 | 1 | 8 | 0 | AT2G34710 | PHB-1D | PHABULOSA 1D |
| GRMZM2G469551 | 8.65 | 3.08 | 4 | 0 | 5 | 0 | AT5G60690 | REV | REVOLUTA |
| GRMZM2G499154 | 0 | 0.04 | 0 | 0 | 0 | 0 | -- | -- | -- |
| GRMZM5G845891 | 0 | 0 | 0 | 0 | 0 | 0 | -- | -- | -- |

WT: W22-ref, MuT: *ago18b::mum*. The values of MuT and WT columns were the RPKM value in RNA-seq. The values of other four columns (4-7) were the reads number in mRNA cRIP-seq. The last three columns were gene annotation to *Arabidopsis thaliana*.
